# Investigation of Quinoa Seeds Fractions and Their Application in Wheat Bread Production

**DOI:** 10.3390/plants10102150

**Published:** 2021-10-11

**Authors:** Ionica Coţovanu, Mădălina Ungureanu-Iuga, Silvia Mironeasa

**Affiliations:** 1 Faculty of Food Engineering, Ştefan cel Mare University of Suceava, 13th Universităţii Street, 720229 Suceava, Romania; ionica.cotovanu@usm.ro; 2 Integrated Center for Research, Development and Innovation in Advanced Materials, Nanotechnologies and Distributed Systems for Fabrication and Control (MANSiD), Ştefan cel Mare University of Suceava, 13th University Street, 720229 Suceava, Romania

**Keywords:** quinoa seed fractions, particle size, wheat bread, addition level

## Abstract

The present study aimed to investigate the influence of quinoa fractions (QF) on the chemical components of wheat flour (WF), dough rheological properties, and baking performance of wheat bread. The microstructure and molecular conformations of QF fractions were dependent to the particle size. The protein, lipids, and ash contents of composite flours increased with the increase of QF addition level, while particle size (PS) decreased these parameters as follows: Medium ˃ Small ˃ Large, the values being higher compared with the control (WF). QF addition raised dough tenacity from 86.33 to 117.00 mm H_2_O, except for the small fraction, and decreased the extensibility from 94.00 to 26.00 mm, while PS determined an irregular trend. The highest QF addition levels and PS led to the highest dough viscoelastic moduli (55,420 Pa for QL_20, 65245 Pa for QM_20 and 48305 Pa for QS_20, respectively). Gradual increase of QF determined dough hardness increase and adhesiveness decrease. Bread firmness, springiness, and gumminess rises were proportional to the addition level. The volume, elasticity, and porosity of bread decreased with QF addition. Flour and bread crust and crumb color parameters were also influenced by QF addition with different PS.

## 1. Introduction

Recently, there has been an increasing emphasis on a healthier lifestyle and healthy eating habits. Refined wheat flour has lower nutritional value: fewer fibers, vitamins, minerals, and phytochemicals than whole-grain wheat flour [1]. Bakery products from refined wheat flour are considered to be nutritionally poor, as the wheat proteins are deficient in essential amino acids such as lysine, tryptophan, and threonine [2,3]. The partial replacement of refined wheat flour with flours made from different crops rich in bioactive compounds has become a necessity during the last years. 

Quinoa (*Chenopodium quinoa* Willd.) is an endemic grain that has attracted much attention in recent times due to its health and wellness benefits [4]. This species, is part of the *Chenopodiaceae* family and has been cultivated for centuries in the Andean countries of Peru and Bolivia [5]. Quinoa reveals a lack of gluten and plays a big role in the human diet because it covers half of people’s daily energy and protein needs [6]. Quinoa is a complete food, being a source of proteins with high biological value, carbohydrates with a low glycemic index, high-quality oil, vitamins (thiamine, riboflavin, niacin, and vitamin E), minerals (magnesium, potassium, zinc, and manganese), and bioactive compounds (dietary fiber, phytosterols, polyphenols and flavonoids) [7,8,9]. Amino acids from quinoa are represented by lysine (twice than wheat), histidine, isoleucine, leucine, tryptophan, and aromatic amino acids (phenylalanine and tyrosine) [8,10]. Quinoa seeds have three main storage compartments (from center to edge): a large central perisperm surrounded by a peripheral embryo or germ, and an endosperm [11]. The starch granules are stored mainly in the perisperm which constitutes almost 40% of the seed mass, while protein, lipids, and minerals are found mostly in the embryo and endosperm [11,12]. Knowing the quinoa seeds morphology is very important for obtaining different enriched fractions because the quality of the baked products is interdependent on the constituents and the particle size of the flour used. Particle size modified the hydration properties of flour and influenced the dough’s rheological properties [13]. 

The dynamic rheological testing methods indicate the viscoelastic behavior of the WF-QF dough given by the interactions between quinoa fractions and wheat gluten network. QF addition caused modifications of the rheological and textural parameters of wheat dough as a result of the gluten dilution [14,15,16]. The ingredients rich in fibers and starch could impact the gas retention of dough, leading to lower bread volume, porosity and elasticity, and higher firmness [17,18], the changes’ magnitude depending on the addition level and particle size. Xiaoxuan et al. [19] demonstrated that the addition of whole quinoa to wheat bread resulted in a decrease of the final product specific volume, while the texture parameters in terms of hardness and chewiness where not significantly influenced at addition levels smaller than 20%. Another study conducted by Calderelli et al. [20] showed that wheat bread enriched with quinoa flour was acceptable from a sensory point of view and presented a high content of protein. Stikic et al. [21] reported positive effects of quinoa flours on the rheological characteristics of wheat dough, while bread-specific volume decreased slightly. According to the results obtained by El-Sohaimy et al. [22], the addition of quinoa in flat bread dough had a slight effect on the rheological characteristics, but did not determine dough deformation. Kurek and Sokolova [17] stated that wheat bread porosity increased due to the protein content of quinoa flour added. The same authors reported that the interaction between particle size and quinoa flour level showed a significant influence on wheat bread chemical and textural properties, particle size having a crucial effect on firmness parameter [17]. In the study of Xu et al. [23], it was demonstrated that the baking performance of wheat bread was not significantly affected by 5% quinoa addition level, while at levels higher than 10% smaller specific volume, increased hardness, and coarse porosity was observed due to the changes of gluten secondary structure and gluten dilution effect.

QF incorporation may impact dough rheology, which could provide information about its processability and hence could predict the baked good quality. The evaluation of wheat flour replacement with QF particle sizes at different addition levels can be useful for enhanced baked goods development, for choosing the appropriate recipes and manufacturing techniques. There are few studies on quinoa-wheat dough proprieties, but no previous studies concerning how the variation in particle size can influence the physico-chemical characteristics, complete texture profile, and dynamic rheological properties have been carried out until now. This work aimed to study the effect of wheat-quinoa composite flour, mix obtained with three different QF particle sizes and four addition levels in wheat flour on dough rheology and bread quality, in relation to the microstructure, molecular conformation, and physico-chemical characteristics of the raw materials.

## 2. Materials and Methods

### 2.1. Materials 

The research was performed on wheat flour type 650 (WF) (harvest 2020) obtained from S.C. MOPAN S.A. (Suceava, Romania), which showed the following characteristics: 14.0% moisture, 12.60% protein, 1.40% fat, 0.65% ash, wet gluten 30%, gluten deformation index 6 mm, and falling number 312.0 s. White quinoa seeds were provided by the SANOVITA (ECUADOR) and were characterized by: moisture content 13.28%, fat content 5.61%, protein content 14.12%, and ash content 2.00%, reported to dried substances. Salt (S.C.SANOVITA S.R.L., Vâlcea, Romania) and fresh Saccharomyces cerevisiae yeast (S.C. ROMPAK S.R.L., Pașcani, România) were acquired from the local market.

### 2.2. Quinoa Fractions Preparation

The quinoa seeds were ground separately with Grain Mill grinder (KitchenAid, Whirlpool Corporation, Benton Harbor, MI, USA), then they were sifted 30 min at 70 Hz with a Retsch Vibratory Sieve Shaker AS 200 basic (Haan, Germany) in order to produce three fractions with different particle sizes (PS): large (L > 300 μm), medium (180 > M < 300 μm), and small (S < 180 μm).

### 2.3. Sample’s Formulations

Each fraction of QF, large (L), medium (M), and small (S) at four addition levels (5%, 10%, 15%, and 20%) were mixed for half an hour in a Yucebas Y21 mixer (Izmir, Turkey), resulting in the following samples: QF_5L, QF_5M, QF_5S, QF_10L, QF_10M, QF_10S, QF_15L, QM_15M, QM_15S, QF_20L, QF_20M, and QF_20S. Wheat flour was considered as control.

### 2.4. Physico-Chemical Characterization of the Formulated Flours

The WF-QF flours were characterized in agreement to ICC methods: moisture content (110/1), protein content (105/2), fat content (105/1), ash content (104/1), and andcarbohydrate content which was calculated by difference, as % of dry matter.

Composite flour colors were analyzed in triplicate by using a CR-700 colorimeter (Konica Minolta, Tokyo, Japan). The flour color characteristics analyzed were: L*—lightness/darkness (0: black and 100: white), and the chromatic components a*—intensity of green (−a*) or red (+a*); and b*—the intensity of blue (−b*) or yellow (+b*).

### 2.5. Dough and Bread Manufacturing

Composite flour or WF (0.3 kg), salt (1.8%), and yeast (3%) were used in the bread manufacturing process. Water absorption capacities of the flours were previously tested on the Mixolab device and used in dough preparation. The dough samples were prepared following the biphasic procedure by mixing water, yeast, and half the amount of composite flour for the sourdough development at 30 ± 2 °C and 85% relative humidity (RH) for 2 h in a leavening chamber (PL2008, Piron, Cadoneghe, Padova, Italy). The leavened sourdough, other half part of WF-QF flour, and salt were kneaded for 10 min with a Kitchen Aid mixer (Whirlpool Corporation, Benton Harbor, MI, USA) and leavened at 30 ± 2 °C and 85% relative humidity (RH) for another 60 min in the same leavening chamber [24]. When fermentation was finished, the dough was divided into 400 g pieces, molded by hand, and leavened in aluminum trays for another 60 min (30 ± 2 °C and 85% RH). The leavened dough was baked at 220 ± 5 °C for 25 min in an oven (Caboto PF8004D, Cadoneghe, Padova, Italy).

### 2.6. Evaluation of Flours Microstructure

The microstructures of WF and QF fractions were evaluated trough electronic scanning microscopy by using a VEGA II LSH device (Tescan, Brno, Czech Republic), at an acceleration tension of 30 kV. The samples were fixed on double-sided adhesive carbon bands and the images were collected at 2000×, 1000×, 500×, and 100× magnifications.

### 2.7. Flours ATR FT-IR Spectra Collection

The ATR FT-IR spectra of WF and QF fractions were collected in triplicate from 650 to 4000 cm−1 wavenumbers on a Thermo Scientific Nicolet iS20 (Waltham, MA, USA) device, at a resolution of 4 cm^−1^ by 32 scans. The molecular characteristics were identified according to previous data from the literature [25,26,27], by using OMNIC software.

### 2.8. Empirical Dough Rheology and Texture Profile Analysis

The viscoelastic behavior of WF-QF flour was determined on a Chopin Alveograph NG (La Garenne Cedex, France) following the standard method SR EN ISO 27971:2009. Each Alveograph curve was analyzed for the following parameters: P (dough resistance), L (dough extensibility), G (swelling index), W (baking strength), and P/L ratio [28].

Texture profile analysis (TPA) was performed on a TVT-6700 texture analyzer (Perten Instruments, Hägersten, Sweden), following the procedure of Mironeasa, Iuga, Zaharia, and Mironeasa [29] with slight modifications. A 3.5 cm stainless steel cylindrical probe was used in a twice-compression test to compress the 50 g of sample up to 50% of its depth, at a test speed of 5.0 mm/s, trigger force of 20 g, and the interval time between two compressions was 12 s. Hardness, adhesiveness, springiness, and cohesiveness were recorded. The measurements were carried out in triplicate.

### 2.9. Fundamental Dough Rheology

A preliminary stress sweep test was performed to identify the limits of the linear viscoelastic region (LVR) in the samples in which increasing strain was applied, from 0.00 to 100 Pa, at constant oscillation frequency of 1 Hz, according to some indications [30]. The dough samples prepared without yeasts, at optimum water absorption capacity, were placed in a measuring system of a HAAKE MARS 40 rheometer (Thermo-HAAKE, Karlsruhe, Germany) with a parallel plate-plates geometry and rested for 5 min prior to testing [31]. The excess dough was removed and a layer of vaseline was applied to the exposed edge to protect it from loss of moisture.

A frequency sweep test from 0.01 to 20 Hz at 10 Pa stress, in the LVR, was applied to determine dough storage (G′) and loss modulus (G″), at 20 °C.

A temperature sweep test was performed at a constant strain of 0.10% and a frequency of 1 Hz, the dough being heated from 20 to 100 °C at a rate of 4.0 ± 0.1 °C per min. The storage (G′) and loss modulus (G″) were recorded as a function of temperature by using Rheowin software. The maximum gelatinization temperature (T_max_) was considered at the maximum G′ value.

### 2.10. Physical Properties of Bread

Bread physical properties were measured in triplicate two hours after baking, in agreement to the Romanian standard procedure SR 91:2007 in terms of volume, porosity, elasticity, and color. Loaf-specific volume (cm^3^) was found by employing the seed displacement procedure. Porosity was calculated based on a sample cylinder volume (60 mm height and 45.50 mm diameter). Elasticity was determined on a crumb cylinder that was pressed for 1 min until half of its height, and then left to recover for 1 min [32].

Color analysis was determined after bread cutting in half and the crumb and the crust color were measured in triplicate by using a CR-700 colorimeter (Konica Minolta, Tokyo, Japan). The bread color characteristics analyzed were L*, a*, and b*. 

### 2.11. Bread Texture Parameters Determination

The bread was cut into slices of 50 mm thickness for the texture properties determination (in triplicate) by using a TVT-6700 texture analyzer (Perten Instruments, Hägersten, Sweden). A 2.5 cm cylindrical stainless-steel probe was used to compress the sample twice to a penetration distance of 20% of its depth, at a test speed of 1.0 mm/s, trigger force of 5 g, with an interval of 15 s between compressions. Firmness, springiness, gumminess, and cohesiveness were registered.

### 2.12. Statistical Analysis

Statistical software SPSS 25.0 (trial version) (IBM, New York, NY, USA) was used to calculate the means and standard deviations for all parameters. Statistically significant differences between parameters were determined by two-way analysis of variance with Tukey’s test at *p* ≤ 0.05 significance level. A principal component analysis (PCA) was applied to observe the relationships between the WF-QF flour chemical constituents, dough rheological measurements, and bread characteristics.

## 3. Results and Discussions

### 3.1. Microstructure of Flours

**Figure 1:**
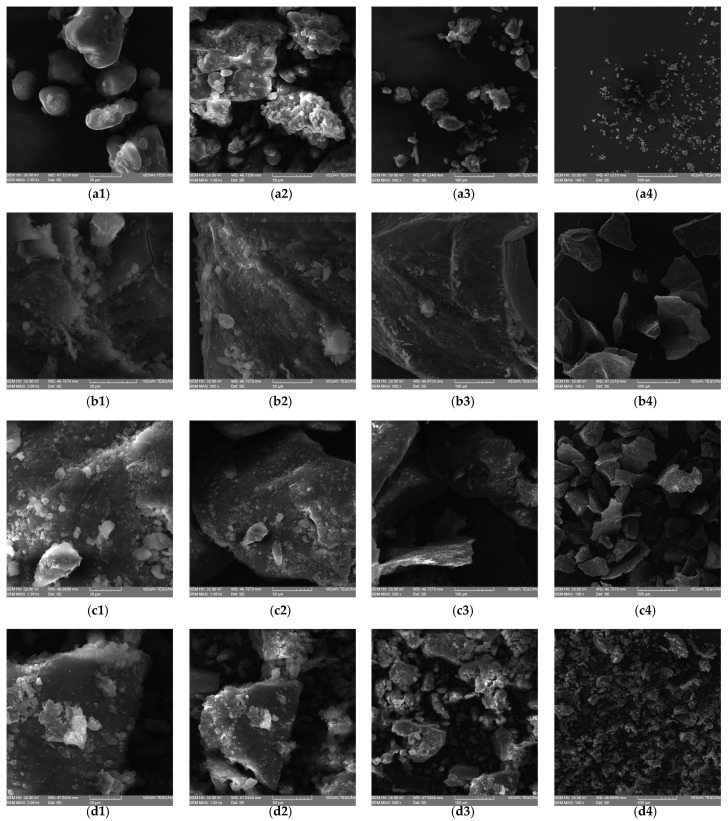
Microstructure of wheat flour (**a1**–**a4**) and quinoa flours fraction L (**b1**–**b4**), fraction M (**c1**–**c4**), and fraction S (**d1**–**d4**) at different magnifications.

The microstructure of QF fractions and WF at different magnifications is presented in Figure 1.

WF structure was composed of starch grains surrounded by gluten proteins (Figure 1a). QF fractionation caused the decrease of PS, the particles presenting polygonal, angular or irregular shapes, similar to the results presented by Romano, Masi, Nicolai, Falciano and Ferrantia [33], and by Alvarez-Jubete, Auty, Arendt, and Gallagher [34]. A more uniform structure of quinoa flour was observed in S fraction (Figure 1(d1–d4)) compared with L (Figure 1(b1–b4)) and M (Figure 1(c1–c4)). Starch grains of rounded and lenticular shapes were present in L and M fractions, while in the case of S fraction irregular starch grains were observed, probably due to the damage caused during milling. Quinoa seeds fractionation led to changes in QF structure, depending on the particle size, which could explain the physico-chemical, rheological, and technological characteristics of flour, dough, and bread.

### 3.2. ATR FT-IR Spectra of Flours

**Figure 2:**
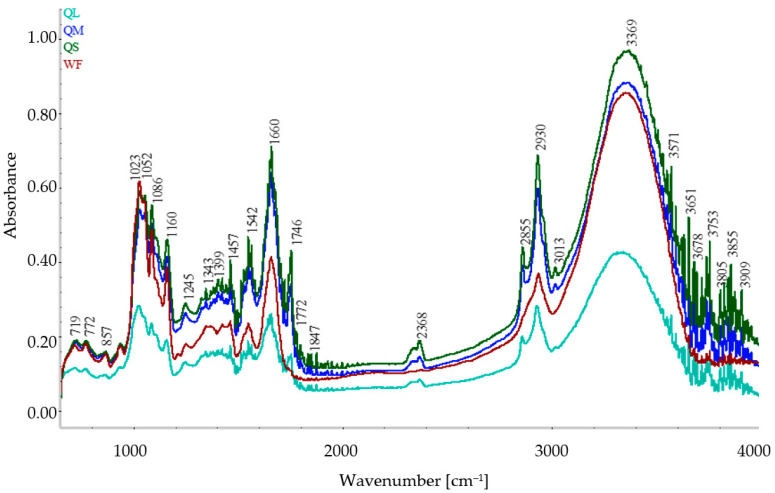
FT-IR spectra of wheat flour and quinoa flours fractions.

The FT-IR spectra of WF and quinoa fractions are shown in Figure 2. There were differences in absorbances between L and M or S quinoa fractions, while the differences between S and M fractions were small. The intensities of the peaks increased as the PS decreased from L < M < S, while WF peaks were between L and M quinoa fractions.

The starch structure identified at about 1100–900 cm^−1^, amide I at 1600–1700 cm^−1^, amide II at 1550 cm^−1^, lipids at 1750 cm^−1^, and 2800–3000 cm^−1^ [26,35] were the most prominent peaks (Figure 2). The band at about 3300 cm^−1^ is given by the stretching vibration of -OH, possibly due to the presence of water, galacturonic acid, arabinose, galactose, xylose, and glucose in quinoa fractions [25]. In the region of 900–1500 cm^−1^, some signals were possibly given by the amylose-lipid complexes, amide III (at 1330–1230 cm^−1^), or carbohydrates such as starch and cellulose [26,36]. The peak observed at 3369 cm^−1^ could be attributed to the O-H stretching vibrations, the band found at 2855 cm−1 could be possibly assigned to the presence of CH2 and CH_3_ groups from aldehydes/ketones [37], while the peak at 1746 cm^−1^ could be attributed to the C=O carbonyl stretching [27]. The stretching given by alcohol and carbonyl groups identified could be possibly due to the chemical structure of quinoa saponins [27]. The band at 2930 cm^−1^ could be given by the stretching vibrations of C-H groups which could be characteristic for polysaccharide-based polymers [25]. The peaks observed at 1660 and 1542 cm^−1^ could give information about the protein amido acids and can reveal modifications in the secondary structure of proteins [37], while at 1086 cm^−1^ possible information about pyranose structure of CH could be found [27]. The peak observed at 1021 cm^−1^ could be attributed to the C-H bending from aromatic structures, similar results being obtained by Czekus et al. [27] for quinoa seeds. The bands present at 857, 772 and 719 cm^−1^ could give information about the substitutions in aromatic rings characterized by aromatic C-H out-of-plane bend [27].

### 3.3. Physico-Chemical Properties of Composite Flours

Table 1 presents the effect of QF addition levels and PS on the composite flour’s physicochemical properties. The studied factors had a significant (*p* < 0.01) effect on chemical parameters of composite flours. The moisture content decreased significantly (*p* < 0.01) with the rise of QF addition and PS decrease, due to the higher wear and possible heat generation that occurs during grinding of smaller PS flours, without prior process conditioning [38]. 

The protein content of composite flours was significantly influenced by QF addition level and increased when the QF addition increased in comparison with the control, due to a higher protein content of quinoa flour than wheat flour [39]. It was noticed that composite flours with medium particles had the highest protein content, followed by flours which contain small particles of QF, while the lower content of protein was found in flours with a large fraction of QF, probably as a result of the botanical structure of quinoa seeds, where proteins and minerals are localized mostly in the embryo and endosperm [12,40] and some part of the grain, richer in proteins, was broken in the form of small particles [41]. Additionally, these variations of protein content in flours enriched with quinoa flour fractions can be explained by the milling equipment used for grinding which differently influenced the structure of the endosperm (hard/soft) and type of endosperm cells (peripheral, prismatic, or central) [15]. Others authors that grounded quinoa seeds with coffee grinder found a higher protein content in small fraction [42]. The lipid content of the blended flours was significantly (*p* < 0.01) affected by the QF addition level and PS compared with wheat flour. The fat content of WF-QF composite flours increased gradually when QF addition level and PS increased, which could be explained by the lipid’s localization in the cells of the endosperm and embryo [11]. The ash content of the wheat-quinoa formulated flours was significantly (*p* < 0.01) affected by the level and PS of QF, and increased with QF addition increase. The variations in ash content could be explained by the cell-wall material from the broken endosperm. Carbohydrate contents significantly (*p* < 0.01) decreased with the rise of QF addition and PS decrease. Similar findings regarding carbohydrates from WF-QF composite flours were found by ElSohaimy et al. [18]. Similar trends of the chemical compositions were previously reported by Coţovanu, Stoenescu and Mironeasa [39], by Ahmed, Thomas and Arfat [42], and Solaesa, Villanueva, Vela, and Ronda [15].

The color parameters, brightness, yellowness, and redness, were significantly (*p* < 0.01) influenced by the QF amount and PS. The lightness L* values decreased in all composite flours when the level of QF increased. The darker composite flour was observed at that was blended with QF medium PS, while the largest PS gave higher lightness of composite flours.

The redness (a*) values significantly (*p* < 0.01) increased with the increase of QF quantity and with PS decrease, which indicates that the flour fraction turned more yellow and whitish and less red. This phenomenon could be explained by the increase of the particle surface area. The yellowness (b*) values decreased when QF addition raised and increased with reducing PS. The yellowness could be explained by the carotenoid pigments [42]. Similar results were found for wheat-quinoa flour blends by Ahmed, Thomas and Arfat [42], and by Demir [43].

### 3.4. Dough Rheological Properties

#### 3.4.1. Alveographic Parameters

The replacement of wheat flour, which contains gluten, is a major technological challenge because gluten is an essential structure-building protein in flour, responsible for the elastic and extensible properties needed to produce good quality bread [44]. The addition level and PS of QF had a significant (*p* < 0.01) effect on the dough’s alveographic properties (Table 2).

QF-WF dough tenacity was statistically (*p* < 0.01) influenced by the addition level, type of QF PS, and the interaction between them. The increment of QF increased gradually with dough tenacity (P) with large and medium PS, while in small PS a decrease was observed. The highest dough tenacity was observed at sample QM_20 and could be possibly explained by the chemical composition of the fraction added. The interactions between the polysaccharides of the fiber and the wheat proteins could be responsible for these increments [45]. The addition of QF in wheat flour dough increased fat and protein content (Table 1), which has an opposite effect; P and W. Sluimer [45] indicated that dough with a low content of lipids is somewhat more flexible, with better machinability, while a high quantity of lipids causes the opposite effect. It can be observed that P increased when wheat flour content decreased, a phenomenon that can be explained by the dough gluten dilution, finding that is consistent with other works [14,46]. PS determined the increase of dough tenacity as follows: S. > L. > M. Dough extensibility index (L) was significantly (*p* < 0.01) affected by the addition level, PS, and their interaction.

A decrement in dough extensibility compared with the control dough was observed with the increased of QF addition level, probably because a preferential pathway for water absorption was created. Large and medium PS determined a decrease of extensibility with size reduction, while the dough with small PS had higher extensibility. The influence of PS on dough extensibility could be correlated with protein content from these PS, which are characterized by minor gluten formation, results which are in line with earlier reports [14]. The index of swelling (G) was significantly (*p* < 0.01) influenced by the factors in the same way as dough extensibility. Dough strength (W) was significantly (*p* < 0.01) affected by both factors, and by the interaction between them. A decrement in the dough strength was observed when QF addition level increased, while an increment was obtained with QF PS reduction, probably due to the addition of a non-gluten flour, which will lead to a gluten dilution and a decrease of its quality and quantity, similar to the results reported by Coţovanu and Mironeasa [24]. As the PS and levels of quinoa flour increased, P increased and L decreased, which resulted in an increase of the P/L ratio from 0.92 to 4.53.

#### 3.4.2. Dynamic Rheological Parameters

Viscoelastic properties of dough play a key role in final products quality and the frequency sweep tests demonstrated that G′ and G″ values were significantly (*p* < 0.01) influenced by the QF addition level, type of PS, and by their interaction. G′ values were greater than G″ values (Figure S1 presented in the Supplementary Materials), so it can be stated that the dough had a viscoelastic behavior. G′ and G″ increased when QF addition level and PS increased. The highest G′ and G″ were observed at large PS which can be explained by the synergistic effect between starch amount and the large PS, the results being in line with those found by Solaesa et al. [15]. Significant differences (*p* < 0.01) between QF dough samples and control were observed in loss tangent (tan δ) regarding QF addition level, although the samples with 5% and 10% addition levels did not show significant differences in this parameter. The decrease of tan δ was proportional with the QF increase for all the tested samples. Significant differences (*p* < 0.01) between the higher PS (L and M) and S were obtained. These variations in rheological properties of the gels could be explained by their different chemical composition (protein, lipid, carbohydrates) and molecular structures (Figure 2), shape, and size of starch granules (Figure 1).

The influence of QF addition level and PS on maximum gelatinization temperature (T_max_) during heating WF-QF composite flour is presented in Table 3. It can be observed a significant (*p* < 0.05) increase of T_max_ with quinoa addition level increase (Figure S2 from the Supplementary Materials), while only L and S fractions significantly affected (*p* < 0.01) this parameter.

An increase in T_max_ values was observed with PS decrease, which may be explained by the high proteins and lipids content, and their low carbohydrates content (which is associated with starch) (Table 1), similar data being reported by Ahmed, Thomas and Arfat [42].

#### 3.4.3. Dough Texture Profile Analysis

**Figure 3:**
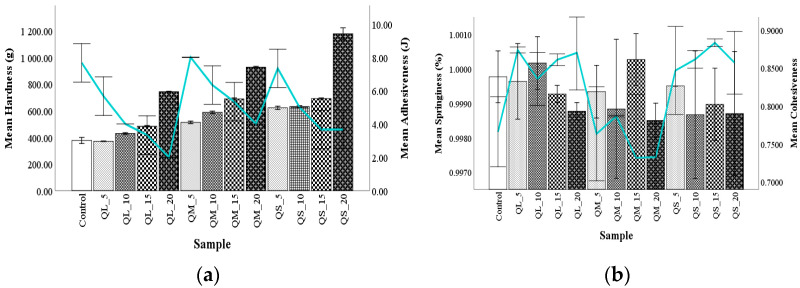
The effect of quinoa flours fractions on dough texture: hardness and adhesiveness (**a**), springiness and cohesiveness (**b**).

The effect of QF addition level and PS on dough texture parameters is shown in Figure 3. Hardness increased with the addition level increase and PS decrease, probably due to 11S-type globulin and 2S albumins, which bound to each other through disulfide bridges and retain more water, than prolamins from wheat flour. This lack of gluten from the dough matrix leads to low gas retention, which forms a harder dough [16]. 

Adhesiveness decreased when the QF addition level increased, which can be explained by the gluten dilution, because it is well known that gliadin has a positive impact on the adhesiveness of the dough [29]. Springiness presented a decrease in comparison with WF dough, but samples QL_10 and QM_15 presented higher values due to the presence of prolamins from wheat flour (40–50%), which can make the wheat flour dough a little bit inelastic, which resulted in the springiness of dough with quinoa fractions that are richer in albumins [22]. Dough cohesiveness values decreased especially in samples formulated with medium PS, which were lower than the control sample and may be explained by the high lipid content from these blends, while for the other samples irregular trends were observed (Figure 3). Wheat gliadins presented a low resistance to extension, which could be responsible for the cohesion of the dough, and wheat glutenins for the dough’s resistance [47].

### 3.5. Physical Properties of Bread

Quinoa flour addition level, its different PS, and the interaction between them significantly (*p* < 0.05) influenced bread characteristics. It is well known that gluten-free grains affect dough gas holding properties which will be negatively reflected in bread volume. QF addition level decreased the volume of bread from 378.13 to 260.00 cm^3^ and from 2.45 to 1.81 cm^3^/g respectively (Table 4), which could be explained by the gluten dilution of doughs with a higher amount of non-gluten flour. Final product quality is strongly influenced by the constituents of the ingredient added. Small fractions have a higher water absorption capacity, but resulted in low bread volume probably due to the intrinsic factors that affect the water-binding properties of flours with a relatively high protein content, factors that refer to amino acid composition, protein conformation, and surface polarity [48]. 

The addition of QF in WF had a significant effect on decreasing the dough strength. Although the WF dough strength was higher, because of its extensibility, the ability to preserve or increase the dough strength absorbed by the dough with the addition of quinoa flours was extremely low [49]. The competition between dietary fiber and starch for water leads to a limited starch swelling and gelatinization, which might be required to reduce the final gas volume fraction in the crumb [50]. Also, the globulins and albumins proteins from quinoa seeds retain more water than wheat protein, which indicates that the gluten network from composite dough was diluted and decreased the alfa amylase activity that affects the proofing process. The results were in line with those obtained previously by Park, Maeda, and Morita [51], Wang et al. [19], and Kurek and Sokolova [17]. Only small PS decreased bread volume significantly (p < 0.01), while no significant differences were observed between the large and medium particles on the volume of bread. This may be related to the higher water absorption capacity of small fractions [17,52]. The porosity and elasticity of WF-QF composite flour were affected significantly (*p* < 0.01) by the QF addition level, type of PS, and interaction between them. Porosity and elasticity decreased with the amount QF added, while PS determined an irregular trend.

The color of composite bread crust was significantly (*p* < 0.01) browner compared with the control (Table 5). The addition level of quinoa flour decreased the lightness (L*) of bread crust which could be due to the color intensity of the raw PS flour and to the dark-colored Maillard reaction products on the crust surface. QF had a higher activity of α–amylase (low falling number) [39] than wheat flour and this could explain the darkness (low L* value) of bread.

Redness (a* value) of the control was lower than bread containing QF, the crust a* values showing significant differences (*p* < 0.01) between samples, increasing when QF addition increased. Yellowness (b* value) increased gradually by increasing the substitution levels of QF. Generally, the results showed that the QF bread samples were darker and redder than the control bread sample which was in accordance with El-Sohaimy et al. [18] and Bilgiçli and İbanoğlu [53]. Carotenoids, chlorophyll, and lignin from quinoa seeds influence the color of flour, crumbs, and crust of the products [54].

Bread crumb brightness was significantly (*p* < 0.01) influenced by the QF addition level and PS. The addition level of quinoa flour in composite flour significantly decreased the lightness (L*) of bread crumb, while the type of PS decreased the crumb lightness as follows: M > S > L. The (a*) redness and (b*) yellowness significantly (*p* < 0.01) raised when the addition level of quinoa flour increased and PS decreased. These results are in line with previous work [18] and can be explained by the higher content of protein in quinoa flour than wheat.

### 3.6. Textural Parameters of Bread

**Figure 4:**
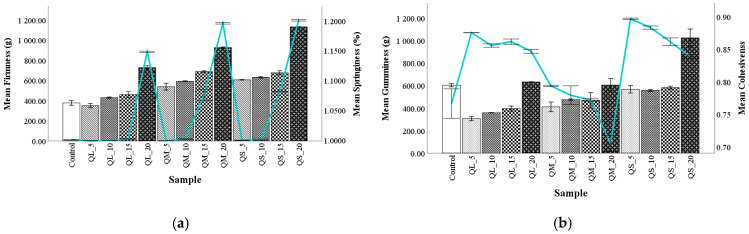
The effect of quinoa flours fractions on bread texture: hardness and springiness (**a**), gumminess and cohesiveness (**b**).

Quinoa flour at different PS and addition levels had significant effects on the bread samples’ texture profiles (Figure 4). Increasing quinoa substitution of wheat flour and interaction of PS and QF significantly increased the firmness of the bread crumb. The bread sample QL_5 presented a lower firmness value than the control bread, which could be due to albumin protein from quinoa seeds, because it can act like gluten in the dough.

It can be observed that all PS at 20% addition significantly increased the firmness of bread, which can be explained by the reduction in the percentage of gluten (responsible for the softness of bread), being also correlated with the high protein content that is found in composite flour which led hard and crunchy bread [18]. The incorporation of 20% QF could cause a negative impact on the acceptability of the bread. This could be related to the significant reduction of air-retention ability and specific volume of the breads [19]. These findings are in line with the results found by Wang et al. [19], El-Sohaimy, Shehata, Mehany, and Zeitoun [22], and by Wolter et al. [55]. Bread springiness followed the same trend as firmness, which raised when more that 10% QF was incorporated. Gumminess increased when QF addition level increased and with the decrease of PS, which may be related to the high content of protein and dietary fiber in quinoa blends, similar results being observed by El-Sohaimy et al. [18]. Cohesiveness in wheat flour and 5% quinoa flour for all PS were slightly higher than in other composite flour, but in general, it decreased with the increase of QF addition and it was higher when PS decreased. These variations could be explained due to the presence of prolamins contained gliadin from wheat flour.

### 3.7. Relations between the Characteristics

The Pearson correlation coefficients (0.56 > r < 0.99) were determined between composite flour chemical constituents, dough rheological and textural parameters, and bread characteristics. Between flour moisture and dough tenacity L (r = 0.75), dough extensibility G (r = 0.76), dough strength W (r = 0.88), dough adhesiveness (r = 0.82), tan δ (r = 0.89), and bread volume (r = 0.97) were found significant positive correlations, while the moisture content was negatively correlated with dough hardness (r = −0.83) and bread firmness (r = −0.82). Probably, in this case, a certain quantity of water enhances the viscoelastic behavior of dough, this amount of water being necessary for protein swelling, the best dough consistency being obtained when enough water is used to swell composite flour components. Similar positive and negative correlations were found between carbohydrates and the parameters listed above.

High positive correlations were found between lipids from flour and dough hardness (r = 0.76) and with bread firmness (r = 0.75). The lipids content from composite flour was negatively correlated with dough extensibility L (r = −0.76), and dough strength W (r = −0.86), dough adhesiveness (r = −0.86), tan δ (r = − 0.84), and with bread elasticity (r = −0.72), volume (r = −0.96), and porosity (r = −0.63). Lipids had a significant influence on bread texture and quality due to their capacity to associate with proteins as they present hydrophilic and hydrophobic groups, and with starch, resulting in starch-lipid complexes [56]. The same trend of positive and negative correlations within these parameters were found with ash content of composite flour. Additionally, a high positive correlation was found between T_max_ and dough extensibility L (r = 0.83), and W (r = 0.76), while negative relationships were found between dough biaxial measurements and G′ and G″. High positive correlations were found between bread volume and L (r = 0.75), G (r = 0.76), W (r = 0.87), dough adhesiveness (r = 0.82), and tan δ (r = 0.88), but a negative relationship between dough rheological parameters and bread physical parameters: dough hardness with bread elasticity (r = −0.74), bread volume, and bread firmness (r = −0.74) were observed. All the correlations listed above were significant at *p* < 0.05. Similar correlation for flour chemical constituents, dough, and bread parameters were found by other authors [57,58].

**Figure 5:**
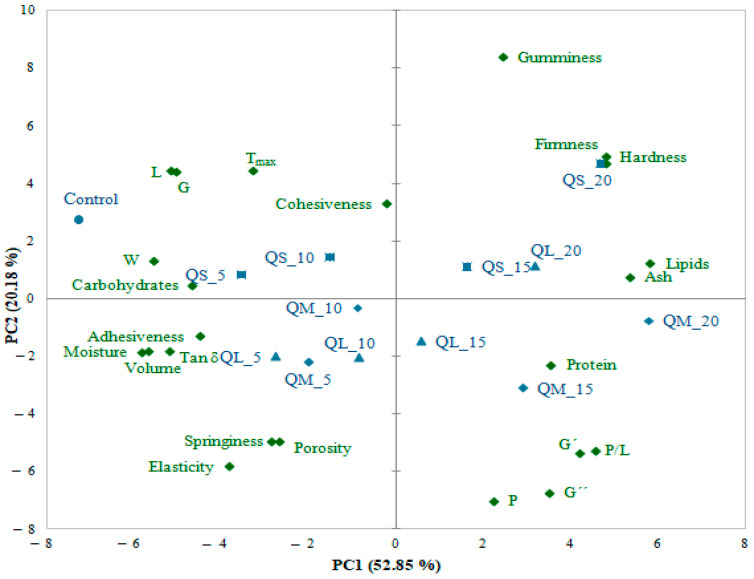
Principal component analysis bi-plot: distribution of the proximate composition, dough rheological and textural parameters, bread physical and textural parameters, and samples.

The principal component analysis (PCA) was used to put in evidence the effect of QF addition and PS on wheat-quinoa composite flour, dough, and bread variables (Figure 5). The two principal components explained 73.03% of the total variance (PC1 = 52.85% and PC2 = 20.18%). The PC1 was associated with composite flour moisture, lipids, ash, carbohydrates, dough alveographic parameters (L, G, W, and P/L), dough textural parameters (hardness, adhesiveness), elastic modulus (G′), tan δ, and bread physical properties (elasticity, volume), while PC2 was associated with dough tenacity (P), viscous modulus (G″) and bread gumminess. It can be observed a high opposition between protein and carbohydrates, P and L alveograph parameters, bread elasticity, and dough hardness.

Regarding bread samples, a good relationship can be observed between the control sample and bread with a 5–10% QF addition level. Samples with medium PS (15–20%) were associated with protein, in opposition to the samples with 20% quinoa large and small fractions which were associated with dough and bread hardness.

## 4. Conclusions

The addition of quinoa flour induced significant changes in dough rheological and textural parameters, bread color, texture, and physical properties, depending on the addition level and particle size used. Quinoa fractionation determined different structural and molecular characteristics, depending on the particle size. The composite flours showed higher chemical components contents in terms of proteins (≥12.47%), lipids (≥1.65%), and ash (≥0.70%), while the carbohydrates content, which varied from 70.20% to 71.25%, was lower compared with the control (71.36%). Higher dough tenacity, hardness and dynamic moduli, and lower extensibility, adhesiveness and dough strength were obtained as the addition level was higher. Bread with raised firmness, springiness and gumminess, was obtained as the quinoa flour level was higher. Bread volume, porosity, elasticity, and luminosity decreased from 378.70 cm^3^ to 260.00 cm^3^, from 72.38% to 57.27%, and from 97.92% to 87.72%, respectively, when QF was added. In order to achieve the highest technological and quality characteristics of bread enriched with quinoa fractions, an optimization of the processing parameters could be performed, taking into account the producers’ conditions and desires. The results presented in this study suggested that the addition levels of 5–15% quinoa fractions to wheat flour could provide acceptable quality characteristics such as rheological behavior which may predict dough handling during processing and final product color, texture, and physical properties. Thus, these results could be of interest for processors in order to develop novel bread formulation with superior characteristics and increased nutritional value.

## Figures and Tables

**Table 1:** Physico-chemical properties of composite flours as affected by quinoa flours fractions addition.

Sample	Moisture (%)	Protein (%)	Lipids (%)	Ash (%)	Carbohydrates (%)		Color		
							L*	a*	b*
Control	14.08 ± 0.08 ^e^	12.45 ± 0.15 ^a^	1.41 ± 0.01 ^a^	0.69 ± 0.04 ^a^	71.36 ± 0.01 ^e^		91.46 ± 0.15 ^d^	-5.13 ± 0.03 ^a^	15.09 ± 0.07 ^b^
QL_5	13.82 ±0.00 ^dy^	12.57 ± 0.03 ^bx^	1.65 ± 0.00 ^by^	0.70 ± 0.00 ^bx^	71.25 ± 0.04 ^dz^		90.87 ± 0.07 ^cz^	−4.89 ± 0.02 ^bx^	14.98 ± 0.05 ^abx^
QL_10	13.64 ± 0.00 ^cy^	12.54 ± 0.06 ^cx^	1.90 ± 0.00 ^cy^	0.76 ± 0.00 ^cx^	71.15 ± 0.06 ^cz^		90.43 ± 0.12 ^bz^	−4.83 ± 0.01 ^cx^	14.51 ± 0.04 ^ax^
QL_15	13.47 ± 0.01 ^by^	12.50 ± 0.09 ^dx^	2.16 ± 0.00 ^dy^	0.81 ± 0.00 ^dx^	71.05 ± 0.11 ^bz^		90.82 ± 0.25 ^bz^	−4.99 ± 0.07 ^cx^	14.50 ± 0.11 ^abx^
QL_20	13.29 ± 0.01 ^ay^	12.47 ± 0.12 ^ex^	2.41 ± 0.00 ^ey^	0.87 ± 0.00 ^ex^	70.95 ± 0.13 ^az^		89.38 ± 0.04 ^az^	−4.60 ± 0.00 ^dx^	14.76 ± 0.02 ^abx^
QM_5	13.80 ± 0.00 ^dxy^	12.91 ± 0.00 ^bz^	1.65 ± 0.00 ^bxy^	0.78 ± 0.00 ^bz^	70.85 ± 0.00 ^dx^		89.35 ± 0.08 ^cx^	−4.85 ± 0.07 ^bx^	14.81 ± 0.13 ^aby^
QM_10	13.60 ± 0.00 ^cxy^	13.22 ± 0.00 ^cz^	1.90 ± 0.00 ^cxy^	0.90 ± 0.00 ^cz^	70.36 ± 0.00 ^cx^		88.64 ± 0.11 ^bx^	−4.72 ± 0.06 ^cx^	14.82 ± 0.12 ^ay^
QM_15	13.47 ± 0.01 ^bxy^	13.54 ± 0.01 ^dz^	2.15 ± 0.00 ^dxy^	1.03 ± 0.00 ^dz^	69.87 ± 0.01 ^bx^		88.10 ± 0.12 ^bx^	−4.69 ± 0.03 ^cx^	15.43 ± 0.52 ^aby^
QM_20	13.21 ± 0.00 ^axy^	13.85 ± 0.03 ^ez^	2.40 ± 0.00 ^exy^	1.16 ± 0.01 ^ez^	69.38 ± 0.02 ^ax^		87.59 ± 0.23 ^ax^	−4.53 ± 0.02 ^dx^	15.33 ± 0.19 ^aby^
QS_5	13.79 ± 0.00 ^dx^	12.75 ± 0.00 ^by^	1.65 ± 0.00 ^bx^	0.74 ± 0.00 ^by^	71.06 ± 0.02 ^dy^		89.80 ± 0.23 ^cy^	−4.93 ± 0.10 ^by^	14.91 ± 0.07 ^aby^
QS_10	13.57 ± 0.03 ^cx^	12.91 ± 0.01 ^cy^	1.89 ± 0.00 ^cx^	0.84 ± 0.00 ^cy^	70.78 ± 0.04 ^cy^		89.05 ± 0.19 ^by^	−4.80 ± 0.02 ^cy^	14.98 ± 0.04 ^ay^
QS_15	13.36 ± 0.05 ^bx^	13.06 ± 0.02 ^dy^	2.14 ± 0.00 ^dx^	0.93 ± 0.00 ^dy^	70.50 ± 0.07 ^by^		88.71 ± 0.13 ^by^	−4.69 ± 0.03 ^cy^	15.22 ± 0.08 ^aby^
QS_20	13.15 ± 0.07 ^ax^	13.22 ± 0.03 ^ey^	2.39 ± 0.00 ^ex^	1.03 ± 0.00 ^ey^	70.20 ± 0.09 ^ay^		88.63 ± 0.05 ^ay^	−4.59 ± 0.05 ^dy^	15.01 ± 0.04 ^aby^
Two-way ANOVA *p* value									
F1:	*p* < 0.01	*p* < 0.01	*p* < 0.01	*p* < 0.01		*p* < 0.01	*p* < 0.01	*p* < 0.01	*p* = 0.01
F2	*p* < 0.01	*p* < 0.01	*p* < 0.01	*p* < 0.01		*p* < 0.01	*p* < 0.01	*p* < 0.01	*p* < 0.01
F1 × F2	*p* = 0.35	*p* < 0.01	*p* = 0.01	*p* < 0.01		*p* < 0.01	*p* < 0.01	*p* < 0.01	*p* < 0.01

F1: level of QF addition; F2: type of particle size; Mean values in the same column with different superscript letters indicates significantly difference (p < 0.05): ^a^–^e^ for QF addition level (0–20%); ^x^–^z^ for QF PS (L, M, and S). L*-lightness; a*-greenness; b*-yelowness.

**Table 2:** Alveographic parameters as affected by quinoa flours fractions.

Sample	P (mm H_2_O)	L (mm)	G	W (×10^−4^ J)	P/L
Control	86.33 ± 0.57 ^a^	94.00 ± 3.00 ^d^	21.55 ± 0.35 ^d^	253.00 ± 4.00 ^d^	0.92 ± 0.03 ^a^
QL_5	88.50 ± 0.50 ^by^	46.50 ± 0.50 ^cy^	15.25 ± 0.05 ^cy^	167.50 ± 2.50 ^cx^	1.80 ± 0.06 ^by^
QL_10	103.50 ± 0.50 ^cy^	39.50 ± 0.50 ^by^	13.80 ± 0.10 ^by^	166.00 ± 0.00 ^cx^	2.65 ± 0.01 ^cy^
QL_15	104.00 ± 1.00 ^cy^	35.50 ± 0.50 ^ay^	12.75 ± 0.05 ^ay^	158.00 ± 2.00 ^bx^	3.39 ± 0.00 ^ey^
QL_20	113.00 ± 1.00 ^dy^	32.00 ± 3.00 ^ay^	12.20 ± 0.60 ^ay^	142.00 ± 5.00 ^ax^	3.21 ± 0.01 ^dy^
QM_5	102.50 ± 1.50 ^bz^	42.00 ± 1.00 ^cx^	14.75 ± 0.15 ^cx^	173.50 ± 0.50 ^cx^	2.44 ± 0.09 ^bz^
QM_10	101.00 ± 0.00 ^cz^	38.00 ± 1.00 ^bx^	14.60 ± 0.00 ^bx^	172.00 ± 1.73 ^cx^	2.35 ± 0.00 ^cz^
QM_15	113.00 ± 3.00 ^cz^	29.00 ± 0.00 ^ax^	12.00 ± 0.00 ^ax^	138.50 ± 4.50 ^bx^	4.53 ± 0.11 ^ez^
QM_20	117.00 ±3.00 ^dz^	26.00 ± 2.00 ^ax^	11.40 ± 0.40 ^ax^	141.50 ± 3.50 ^ax^	3.92 ± 0.37 ^dz^
QS_5	98.50 ± 0.50 ^bx^	58.50 ± 1.50 ^cz^	17.00 ± 0.20 ^cz^	211.50 ± 8.31 ^cx^	1.66 ± 0.01 ^bx^
QS_10	96.00 ± 1.00 ^cx^	53.00 ± 3.00 ^bz^	16.05 ± 0.35 ^bz^	179.50 ± 0.50 ^cx^	1.87 ± 0.03 ^cx^
QS_15	92.00 ± 1.00 ^cx^	43.50 ± 1.50 ^az^	14.60 ± 0.20 ^az^	158.00 ± 2.00 ^bx^	2.22 ± 0.01 ^ex^
QS_20	80.50 ± 1.50 ^dx^	37.00 ± 1.00 ^az^	13.55 ± 0.15 ^az^	117.50 ± 6.50 ^ax^	2.18 ± 0.01 ^dx^
Two-way ANOVA *p* value					
F1	*p* < 0.01	*p* < 0.01	*p* < 0.01	*p* < 0.01	*p* < 0.01
F2	*p* < 0.01	*p* < 0.01	*p* < 0.01	*p* < 0.01	*p* < 0.01
F1 × F2	*p* < 0.01	*p* < 0.01	*p* < 0.01	*p* < 0.01	*p* < 0.01

F1: level of QF addition; F2: type of particle size; means in the same column with different superscripts letters indicate significant difference (*p* < 0.01): ^a^–^e^ for QF addition level (0–20%); and ^x^–_z_ for QF PS (L, M, and S). P—dough tenacity; L—dough extensibility; G—index of swelling; W—dough strength; P/L—curve configuration ratio.

**Table 3:** Dynamic moduli and gelatinization temperatures as affected by quinoa flours fractions.

Type of Sample	G′ at 1 Hz (Pa)	G″ at 1 Hz (Pa)	Tan δ at 1 Hz (adim.)	T_max_ (°C)
Control	26,370 ± 70.15 ^a^	9488 ± 60.00 ^a^	0.3598 ± 0.00 ^d^	82.74 ± 0.49 ^c^
QL_5	44,600 ± 270.00 ^by^	17,125 ± 145.00 ^cy^	0.3839 ± 0.00 ^cy^	78.32 ± 0.05 ^abx^
QL_10	47,150 ± 190.00 ^cy^	15,615 ± 25.00 ^by^	0.3311 ± 0.01 ^by^	78.87 ± 0.02 ^bx^
QL_15	52,790 ± 285.00 ^dy^	19,970 ± 100.00 ^dy^	0.3782 ± 0.00 ^dy^	78.68 ± 0.19 ^abx^
QL_20	55,420 ± 40.00 ^ey^	20,525 ± 535.00 ^dy^	0.3703 ± 0.00 ^dy^	78.97 ± 1.01 ^ax^
QM_5	34,865 ± 525.00 ^bz^	11,240 ± 60.00 ^cz^	0.3223 ± 0.00 ^cy^	78.47 ± 0.05 ^abxy^
QM_10	47,905 ± 615.00 ^cz^	16,935 ± 145.00 ^bz^	0.3535 ± 0.00 ^by^	79.06 ± 0.14 ^bxy^
QM_15	57,440 ± 310.00 ^dz^	18,640 ± 170.00 ^dz^	0.3245 ± 0.00 ^dy^	79.45 ± 0.08 ^abxy^
QM_20	65,245 ± 205.00 ^ez^	19,745 ± 95.00 ^dz^	0.3026 ± 0.00 ^dy^	79.41 ± 0.03 ^axy^
QS_5	31,320 ± 280.00 ^bx^	10,175 ± 335.00 ^cx^	0.3248 ± 0.00 ^cx^	80.28 ± 0.15 ^aby^
QS_10	32,360 ± 200.00 ^cx^	10,853 ± 116.50 ^bx^	0.3353 ± 0.00 ^bx^	80.45 ± 0.18 ^by^
QS_15	39,260 ± 585.00 ^dx^	14,360 ± 420.00 ^dx^	0.3657 ± 0.00 ^dx^	78.74 ± 0.07 ^aby^
QS_20	48,305 ± 240.00 ^ex^	15,725 ± 45.00 ^dx^	0.3255 ± 0.00 ^dx^	78.97 ± 0.11 ^ay^
Two-way ANOVA *p* value				
F1	*p* < 0.01	*p* < 0.01	*p* < 0.01	*p* < 0.01
F2	*p* < 0.01	*p* < 0.01	*p* < 0.01	*p* < 0.01
F1 × F2	*p* < 0.01	*p* < 0.01	*p* < 0.01	*p* < 0.01

F1: level of QF addition; F2: type of particle size; means in the same column with different superscript letters indicate significant difference (*p* < 0.01): _a_–^e^ for QF addition level (0–20%); and ^x^–_z_ for QF PS (L, M, and S). G′—elastic modulus; G′’—viscous modulus; tan δ—loss tangent; T_max_—maximum gelatinization temperature.

**Table 4:** Physical characteristics of bread as affected by quinoa flours fractions.

Sample	Loaf Volume (cm ^3^)	Specific Volume (g/cm ^3^)	Porosity (%)	Elasticity (%)
Control	378.70 ± 1.12 ^e^	2.45 ± 0.00 ^e^	64.33 ± 0.11 ^b^	91.72 ± 0.07 ^b^
QL_5	372.60 ± 0.52 ^dx^	2.25 ± 0.02 ^dxy^	72.38 ± 0.16 ^ex^	97.92 ± 0.37 ^ez^
QL_10	358.87 ± 1.02 ^cx^	2.20 ± 0.00 ^cxy^	67.93 ± 0.05 ^dx^	94.11 ± 0.84 ^dz^
QL_15	335.27 ± 0.37 ^bx^	2.00 ± 0.06 ^bxy^	66.35 ± 0.34 ^cx^	93.17 ± 0.45 ^cz^
QL_20	317.01 ± 1.24 ^ax^	1.93 ± 0.01 ^axy^	57.27 ± 0.52 ^ax^	89.99 ± 1.66 ^az^
QM_5	371.30 ± 1.21 ^dx^	2.24 ± 0.01 ^dy^	72.47 ± 0.07 ^ez^	96.36 ± 0.29 ^eyz^
QM_10	363.53 ± 1.27 ^cx^	2.22 ± 0.01 ^cy^	70.87 ± 0.46 ^dz^	94.51 ± 0.31 ^dyz^
QM_15	338.86 ± 0.15 ^bx^	2.05 ± 0.00 ^by^	67.63 ± 0.81 ^cz^	93.48 ± 0.15 ^cyz^
QM_20	318.63 ± 0.81 ^ax^	1.93 ± 0.00 ^ay^	66.32 ± 0.58 ^az^	89.74 ± 0.50 ^ayz^
QS_5	356.66 ± 1.52 ^dy^	2.21 ± 0.02 ^dx^	71.97 ± 0.52 ^ey^	96.17 ± 0.10 ^exy^
QS_10	347.33 ± 2.08 ^cy^	2.18 ± 0.00 ^cx^	70.51 ± 0.09 ^dy^	94.86 ± 0.93 ^dxy^
QS_15	303.66 ± 3.51 ^by^	2.04 ± 0.03 ^bx^	66.63 ± 0.80 ^cy^	92.00 ± 0.63 ^cxy^
QS_20	260.00 ± 3.00 ^ay^	1.81 ± 0.08 ^ax^	61.60 ± 1.01 ^ay^	87.72 ± 0.96 ^axy^
Two-way ANOVA *p* value				
F1	*p* < 0.01	*p* < 0.01	*p* < 0.01	*p* < 0.01
F2	*p* < 0.01	*p* < 0.01	*p* < 0.01	*p* < 0.01
F1 × F2	*p* < 0.01	*p* = 0.01	*p* < 0.01	*p* = 0.01

F1: level of QF addition; F2: type of particle size; means in the same column with different superscript letters indicate significant difference (*p* < 0.01): ^a^–^e^ for QF addition level (0–20%); and ^x^–^z^ for QF PS (L, M, and S).

**Table 5:** Crust and crumb color parameters of bread samples as affected by quinoa flours fractions.

Sample	Crust Color			Crumb Color		
	L*	a*	b*	L*	a*	b*
Control	67.36 ± 0.19 ^d^	0.78 ± 0.22 ^a^	32.27 ± 0.28 ^cy^	72.30 ± 0.27 ^e^	−4.48 ± 0.03 ^a^	19.02 ± 0.23 ^a^
QL_5	64.99 ± 0.74 ^dx^	4.09 ± 0.30 ^by^	29.65 ± 0.17 ^az^	75.21 ± 0.19 ^dy^	−4.24 ± 0.09 ^bx^	19.75 ± 0.13 ^bx^
QL_10	61.79 ± 0.07 ^cx^	5.29 ± 0.10 ^cy^	32.74 ± 0.20 ^bz^	64.64 ± 1.07 ^cy^	−3.95 ± 0.24 ^cx^	20.09 ± 0.59 ^cx^
QL_15	60.71 ± 0.40 ^bx^	6.64 ± 0.24 ^dy^	34.00 ± 0.78 ^cz^	64.00 ± 0.50 ^by^	−3.86 ± 0.03 ^dx^	21.30 ± 0.56 ^dx^
QL_20	59.88 ± 0.97 ^ax^	6.79 ± 0.53 ^dy^	34.28 ± 0.44 ^dz^	63.37 ± 0.47 ^ay^	−3.67 ± 0.04 ^ex^	21.26 ± 0.10 ^ex^
QM_5	63.36 ± 0.56 ^by^	3.59 ± 0.25 ^bx^	24.56 ± 0.22 ^ax^	69.88 ± 0.73 ^dy^	−4.17 ± 0.14 ^by^	19.54 ± 0.60 ^by^
QM_10	65.48 ± 0.43 ^cy^	4.74 ± 0.38 ^cx^	30.66 ± 0.59 ^bx^	66.96 ± 0.85 ^cy^	−3.78 ± 0.02 ^cy^	21.54 ± 0.22 ^cy^
QM_15	63.62 ± 0.26 ^by^	4.90 ± 0.18 ^dx^	31.32 ± 0.87 ^cx^	65.64 ± 0.38 ^by^	−3.33 ± 0.02 ^dy^	21.89 ± 0.07 ^dy^
QM_20	62.23 ± 0.51 ^ay^	5.11 ± 0.32 ^dx^	32.76 ± 0.69 ^dx^	63.40 ± 0.67 ^ay^	−3.17 ± 0.09 ^ey^	23.50 ± 0.22 ^ey^
QS_5	64.25 ± 0.31 ^dx^	3.54 ± 0.09 ^bx^	29.03 ± 1.14 ^ay^	65.45 ± 1.27 ^dx^	−3.74 ± 0.08 ^bz^	20.42 ± 0.49 ^by^
QS_10	62.01 ± 0.61 ^cx^	4.42 ± 0.20 ^cx^	30.51 ± 0.36 ^by^	65.45 ± 0.33 ^cx^	−3.66 ± 0.04 ^cz^	22.12 ± 0.70 ^cy^
QS_15	60.01 ± 0.74 ^bx^	4.78 ± 0.32 ^dx^	31.71 ± 0.43 ^cy^	65.35 ± 0.51 ^bx^	−3.03 ±0.09 ^dz^	22.78 ± 0.49 ^dy^
QS_20	57.79 ± 0.88 ^ax^	5.11 ± 0.14 ^dx^	34.57 ± 0.41 ^dy^	64.37 ± 1.69 ^ax^	−2.23 ± 0.10 ^ez^	22.19 ± 1.25 ^ey^
Two-way ANOVA *p* value						
F1	*p* < 0.01	*p* < 0.01	*p* < 0.01	*p* < 0.01	*p* < 0.01	*p* < 0.01
F2	*p* < 0.01	*p* < 0.01	*p* < 0.01	*p* < 0.01	*p* < 0.01	*p* < 0.01
F1 × F2	*p* < 0.01	*p* < 0.01	*p* < 0.01	*p* < 0.01	*p* < 0.01	*p* < 0.01

F1: level of QF addition; F2: type of particle size; means in the same column with different superscript letters indicate significant difference (*p* < 0.01): ^a^–^e^ for QF addition level (0–20%); and ^x^–^z^ for QF PS (L, M, and S). L*, a*, b*: CIELAB color parameters.

## Data Availability

Not applicable.

## Supplementary Materials

The following are available online at https://www.mdpi.com/article/10.3390/plants10102150/s1, Figure S1: Variations of elastic (G′) and viscous modulus (G″) with frequency for wheat-quinoa fractions with large (L), medium (M), and small (S) particle sizes dough with: 5% (a), 10% (b), 15% (c), and 20% (d) addition level; Figure S2: Variations of elastic (G′) and viscous modulus (G″) with temperature for wheat-quinoa fractions with large (L), medium (M), and small (S) particle sizes dough with: 5% (a), 10% (b), 15% (c), and 20% (d) addition level.
