# Supplementary material for: Investigation of Quinoa Seeds Fractions and Their Application in Wheat Bread Production
Source: Plants (Basel). 2021 Oct 11;10(10):2150. doi: 10.3390/plants10102150 (PMC8540254; doi:10.3390/plants10102150)
Supplement: Supplementary file 1 [file plants-10-02150-s001.zip › plants-1391171-supplementary.pdf]

Supplementary file

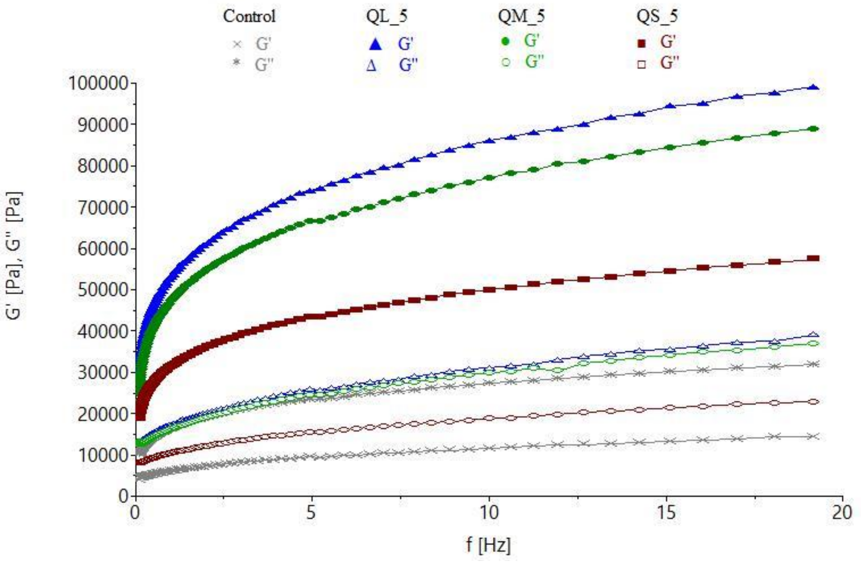

a

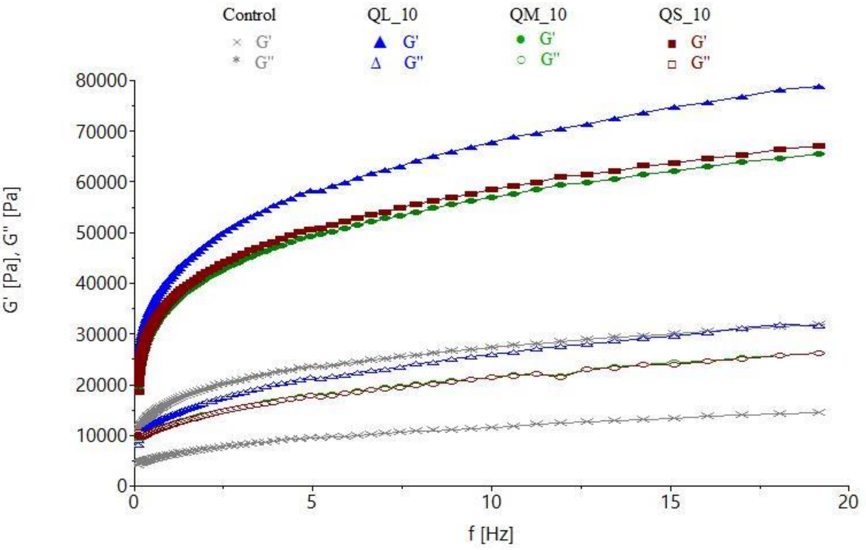

b

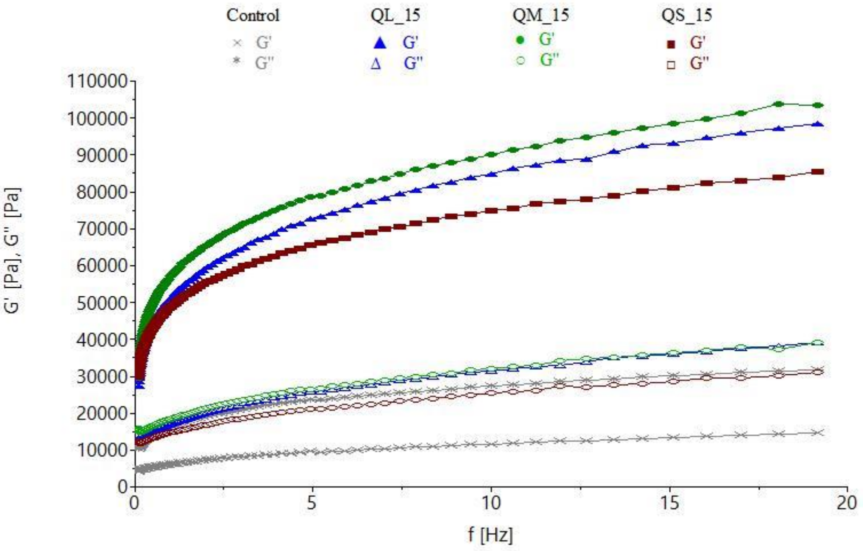

c

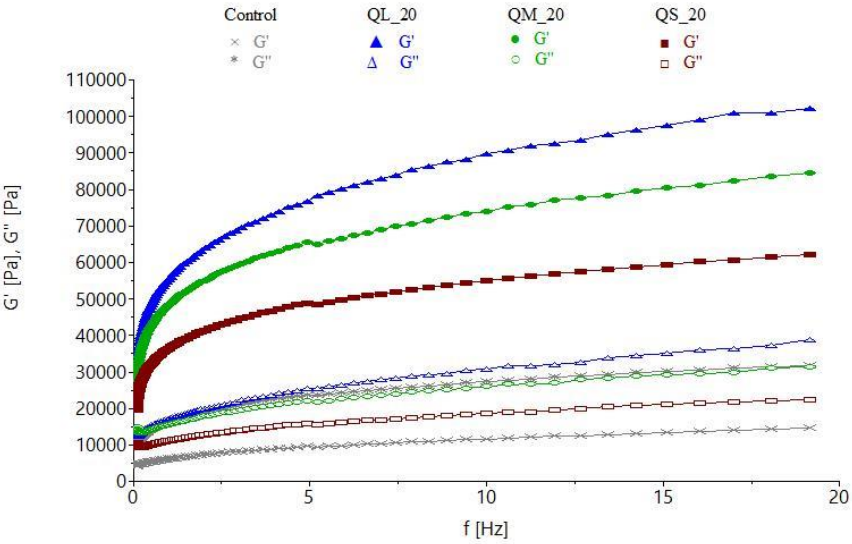

d

Figure S1. Variations of elastic (G’) and viscous modulus (G’’) with frequency for wheat-quinoa fractions with large (L), medium (M), and small (S) particle sizes dough with: 5% (**a**), 10% (**b**), 15% (**c**), and 20% (**d**) addition level

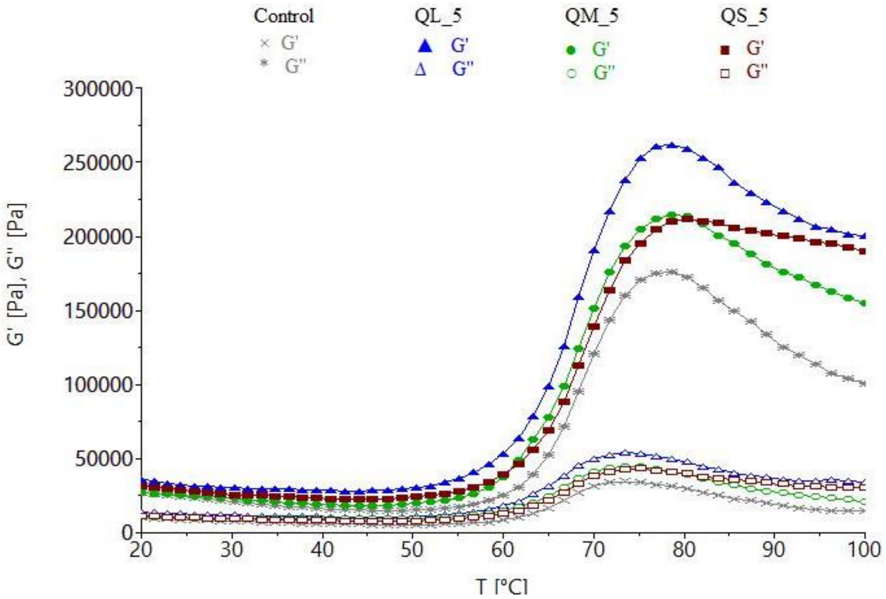

a

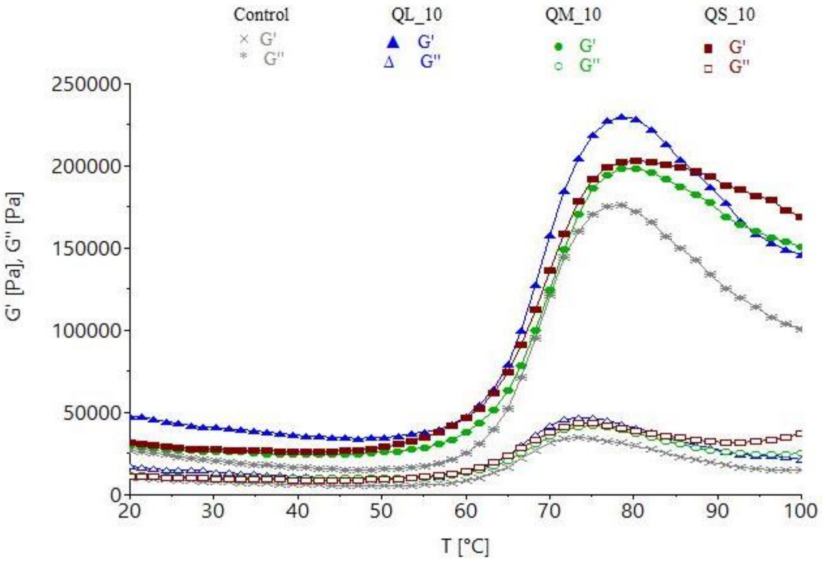

b

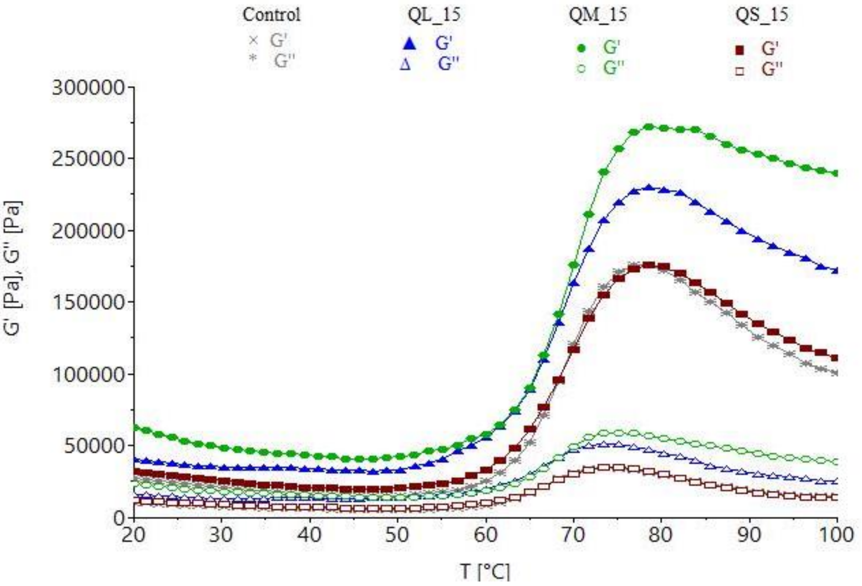

c

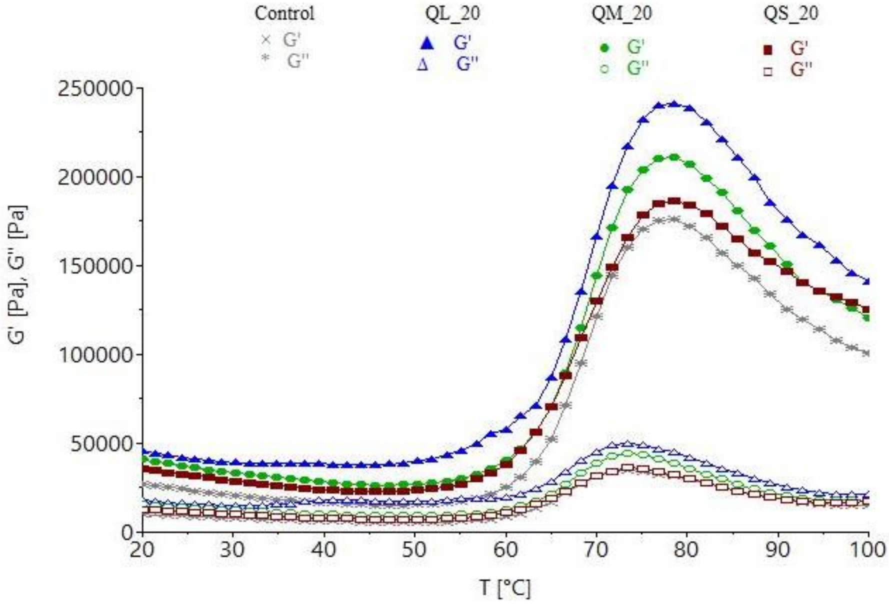

d

Figure S2. Variations of elastic (G′) and viscous modulus (G″) with temperature for wheat-quinoa fractions with large (L), medium (M), and small (S) particle sizes dough with: 5% (**a**), 10% (**b**), 15% (**c**), and 20% (**d**) addition level
